# Divergent suicidal symptomatic activations converge on somato-cognitive action network in depression

**DOI:** 10.1038/s41380-024-02450-7

**Published:** 2024-02-14

**Authors:** Jiao Li, Dajing Wang, Jie Xia, Chao Zhang, Yao Meng, Shuo Xu, Huafu Chen, Wei Liao

**Affiliations:** 1 The Clinical Hospital of Chengdu Brain Science Institute, School of Life Science and Technology, University of Electronic Science and Technology of China, Chengdu, 611731 P.R. China; 2 MOE Key Lab for Neuroinformation, High-Field Magnetic Resonance Brain Imaging Key Laboratory of Sichuan Province, University of Electronic Science and Technology of China, Chengdu, 611731 P.R. China

**Keywords:** Depression, Diagnostic markers

## Abstract

Individuals with depression have the highest lifetime prevalence of suicide attempts (SA) among mental illnesses. Numerous neuroimaging studies have developed biomarkers from task-related neural activation in depressive patients with SA, but the findings are inconsistent. Empowered by the contemporary interconnected view of depression as a neural system disorder, we sought to identify a specific brain circuit utilizing published heterogeneous neural activations. We systematically reviewed all published cognitive and emotional task-related functional MRI studies that investigated differences in the location of neural activations between depressive patients with and without SA. We subsequently mapped an underlying brain circuit functionally connecting to each experimental activation using a large normative connectome database (*n* = 1000). The identified SA-related functional network was compared to the network derived from the disease control group. Finally, we decoded this convergent functional connectivity network using microscale transcriptomic and chemo-architectures, and macroscale psychological processes. We enrolled 11 experimental tasks from eight studies, including depressive patients with SA (*n* = 147) and without SA (*n* = 196). The heterogeneous SA-related neural activations localized to the somato-cognitive action network (SCAN), exhibiting robustness to little perturbations and specificity for depression. Furthermore, the SA-related functional network was colocalized with brain-wide gene expression involved in inflammatory and immunity-related biological processes and aligned with the distribution of the GABA and noradrenaline neurotransmitter systems. The findings demonstrate that the SA-related functional network of depression is predominantly located at the SCAN, which is an essential implication for understanding depressive patients with SA.

## Introduction

Depression is a common mental illness, with approximately 280 million people affected around the world [1]. A systematic review indicated that depressive patients have a higher risk of suicidality than individuals without depression [2]. Furthermore, the lifetime mortality rate due to suicide in patients with depression reaches 15% [3]. Suicide attempts (SA) are an important factor contributing to mortality [4]. Thus, identifying the underlying brain circuits in depressive patients with SA could help us understand the relevant neurological basis and ultimately prevent suicide.

Considerable efforts have been dedicated to elucidating neural mechanisms in depressive patients with SA during various cognitive and emotional tasks [5, 6], including the Balloon Analog Risk Task [7], the facial emotion processing task [8–10], the Go/NoGo task [11], the Iowa Gambling task [12], the discounting task [13], and the Cyberball Game [14]. However, loci of neural activation are plagued with low reproducibility. For instance, Pan et al. found that depressive patients with SA exhibited significantly increased activation in the right anterior cingulate gyrus, left dorsolateral prefrontal cortex, primary sensory cortex, and right middle temporal gyrus during a facial emotional task compared with depressive patients without SA [10]. However, Chase et al. did not find different activations between depressive patients with SA and without SA during the same task [15].

The conventional activation likelihood estimation (ALE) meta-analysis technique [16] has been used to identify commonality amidst the heterogeneities to solve the “reproducibility crisis” in neuroimaging [17, 18]. However, reproducibility in ALE meta-analysis is mainly focused on whether the same brain regions are activated, ignoring the connectivity among activated regions. Recently, there has been a shift in the localization of psychiatric diseases from isolated brain regions to shared brain networks [19, 20]. Cash and colleagues [20] identified a robust and clinically significant distributed brain network of depression that was irreconcilable with the ALE method using a task-related database. Considering depression as a neural system disorder by the contemporary interconnected view [21] and the functional integration between different brain regions during psychological processes [22], we hypothesized that heterogeneous abnormal neuroimaging activations in depression with SA could map to a common brain network.

To uncover the common brain network underpinning SA in depressive patients by the abnormal activation coordinates during task performance, we chose a recently proposed method termed “activation network mapping” (ANM) [23], which was developed from the “lesion network mapping” technique [24]. The ANM method replaces brain lesions with reported activation coordinates as input to obtain the brain network of specific symptoms based on a large cohort of resting-state normative functional connectivity. The ANM technique has been used to derive brain networks for schizophrenia with the symptoms of formal thought disorder and auditory verbal hallucinations [25]. Similar to ANM, previous studies have used the coordinate-based network mapping technique to identify networks associated with depression and late-life depression using brain structure differences [19] and a shared brain network associated with Parkinson’s disease with dementia from neuroimaging findings [26]. Overall, the ANM method integrates the heterogeneous neuroimaging data to identify a common network in depression with SA from task-based functional MRI (fMRI) studies.

In this study, we aimed to identify the corresponding brain circuit in depressive patients with SA using the ANM method. We first tested whether the heterogeneous abnormal brain activations in depressive patients with SA across multiple experimental tasks would localize to a common network. Then, we tested whether this identified network was robust to perturbations of analyzed parameters and experimental tasks and was specific in depression compared with the disease control group. Finally, we decoded the identified brain network by microscale transcriptomic and chemo-architectures and macroscale psychological processes.

## Methods

### Search strategy

This systematic meta-analysis was conducted according to the Preferred Reporting Items for Systematic Reviews and Meta-Analyses (PRISMA) standard [27].

The search for neuroimaging studies investigating functional alterations in depressive patients with SA compared to those without SA was performed up to 1 October 2022 on PubMed and Web of Science. Keywords restricted to human studies were: (suicide OR “suicide*”) AND (fMRI OR “Functional MRI” OR “Functional MRI” OR “Functional Magnetic Resonance Imaging”) AND (depression OR MDD OR “major depression” OR “major depressive disorder” OR “depressive disorder” OR depressed). Other sources included reference lists of previous reviews and meta-analysis articles that summarized the studies about task-related alterations in depressive patients with SA.

### Data extraction

The inclusion criteria were as follows: (i) specific tasks were performed during fMRI scanning; (ii) depressive patients without SA were included; (iii) whole-brain activations were compared between depressive patients with and without SA; (iv) the Talairach or Montreal Neurological Institute (MNI) coordinates of significant brain regions were reported; (v) studies did not include other mental illness patients other than depressive patients. The following exclusion criteria were used: (i) specific regions of interest were analyzed, or the brain was only partially covered; (ii) no significant differences between depressive patients with SA and without SA were reported; (iii) the coordinates were not reported; (iv) differences in functional connectivity rather than activation were examined.

Information was extracted from each identified study, including author, publication year, experimental tasks, sample size, demographics, clinical scale score, and coordinates. The Talairach coordinates were converted into the MNI coordinates.

### Activation network mapping

The ANM method [23] was used to test whether heterogeneous abnormal activation regions in depression with SA localize to a common brain network. This method is similar to coordinate-based network mapping [28]. *Resting-state functional connectivity (RSFC) analysis section*: First, a 4-mm-radius sphere at each reported coordinate was created. Spheres from the same experimental task were merged to produce a combined seed. Eleven combined seeds were generated for subsequent analyses. Second, functional connectivity was calculated between each combined seed and voxels at the whole-brain level for each subject based on a publicly available resting-state connectome database [29, 30], i.e., Brain Genomics Superstruct Project (https://dataverse.harvard.edu/dataverse/GSP). Third, each subject-level correlation r-map (above 1000) was transformed to a Fisher z-map. Then, the experiment-level t-map was generated by comparing all the 1000 subject-level Fisher z-maps from the same experimental task against zero using the one-sample *t* test. *Overlapping section*: First, each experiment-level t-map was thresholded at t > 3 (corresponding to voxel-wise false discovery rate [FDR] correction with *P* < 0.01). Second, all binarized maps were overlapped and thresholded at 60% [23] to create a group-level network overlap map, named SA-related ANM in depression. Several control analyses were performed to test the robustness of the identified SA-related ANM in depression (Supplementary Methods).

### Specificity analysis of the SA-related ANM in depression

To assess the specificity of the SA-related ANM in depression, we explored SA-related ANM in patients with bipolar disorder (BD) and schizophrenia (SCZ) (hereafter termed “disease control group”) using the same methods and parameters as in depression patients. Four experimental tasks were identified from three articles, which explored the brain activity differences between BD or SCZ patients with SA and without SA. Considering the limited number of studies in BD and SCZ patients with SA, the two disorders were defined as one disease control group. A priori-defined region of interest (ROI) in the insula, obtained from a meta-analysis that integrated the task-related brain activation abnormalities in depression with SA [31], was used to test the specificity of the SA-related ANM in depression. All experiment-level t-statistics were converted to z-statistics using the t2z function in SPM12. Group mean z-maps were separately calculated for patients with depression and the disease control group. Next, the voxels’ z-values within the insula were extracted from mean z-map for each group. Finally, differences of z-value distribution were evaluated between the depression and disease control groups using Cohen’s d test. Meanwhile, the precentral and postcentral gyri were used as the post-hoc ROI to test the specificity of SA-related ANM in depression. The priori-defined and post-hoc ROIs were generated from the AAL3 brain atlas [32].

### Transcriptomic analysis

The Allen Human Brain Atlas dataset [33] (https://human.brain-map.org/) allows us to investigate the relationship between SA-related ANM in depression and transcriptome. Gene expression data of brain tissue samples were collected from six postmortem human brains. The microarray expression data were prerocessed by the *abagen* toolbox [34], which provides reproducible workflows to process gene expression data following an established practical guide [35].

After preprocessing gene expression data using the *abagen* toolbox with the Human Brainnetome atlas with 246 regions [36], we obtained a regional expression matrix for each donor with 246 rows corresponding to brain regions, and 15,633 columns corresponding to the retained genes. To get stably expressed genes, we selected the genes with a mean similarity across brain regions above 0.1 between donors, resulting in 12,824 genes. We then averaged the retained genes across all donors. Because only two donors have gene expression in the right hemisphere, we extracted the left hemisphere gene expression data for the following analyses. Consequently, we generated a 123 × 12,824 matrix describing the transcript levels of the left hemisphere.

Partial least squares (PLS) correlation analysis [37] was used to determine the relationship between SA-related ANM in depression and the gene expression patterns of all 12,824 genes. The SA-related ANM in depression was firstly downsampled to 246 regions, i.e., the averaged value of all voxels within the region as the region’s value. Gene expression data were used as predictor variables, and SA-related ANM in depression were used as the response variables in PLS correlation. The first component of PLS (PLS1) was strongly correlated with SA-related ANM in depression across 123 regions. To correct the spatial autocorrelation, we test the statistical significance of variance explained by randomizing the response variables 5,000 times using the Moran spectral randomization [38, 39]. A bootstrapping method was used to estimate each gene’s weighting coefficient in the PLS analysis. The ratio of the weight of each gene to its bootstrap standard error (5000 times) was utilized to calculate z scores of each gene weight on PLS1 [40]. Genes were ranked according to their contribution to PLS1.

The gene enrichment analysis was conducted by Metascape (https://metascape.org/), which can perform gene meta-analysis with over 40 independent knowledge bases [41]. Genes with PLS1 weights of z > 3.69 or z < −3.69 (all *P*_FDR_ < 0.001) were input into the Metascape website to perform Kyoto Encyclopedia of Genes and Genomes (KEGG) and Gene Ontology (GO) biological processes analyses with the background of 16,831 brain-expressed genes [42]. The background genes were obtained by excluding probes if they did not exceed the background in at least 20% of samples after probe to gene re-annotation. The obtained biological processes were corrected by FDR correction (*P* < 0.05).

### Neurotransmitter receptors and transporters analysis

We assessed the relationships between SA-related ANM in depression and neurotransmitter systems across all brain regions. To this end, we obtained whole-brain volumetric PET receptor images from a cohort of over 1200 healthy individuals to construct an overview of 19 distinct neurotransmitter receptors and transporters across nine different neurotransmitter systems [43] (https://github.com/netneurolab/hansen_receptors/tree/main/data/PET_nifti_images). Based on the previous study [44], five neurotransmitters associated with depression were analyzed in this study, including serotonin (5-HT_1a_, 5-HT_1b_, 5-HT_2a_, 5-HT_4_, 5-HT_6_, and 5-HTT), noradrenaline (NAT), dopamine (D_1_, D_2_, and DAT), glutamate (mGluR_5_ and NMDAR), and gamma-aminobutyric acid (GABA_A_). Similar to the procedures in Hansen et al., receptors and transporters with more than one mean image of the same tracer (i.e., 5-HT_1b_, D_2_, and mGluR_5_) were averaged together in a manner that weights each image by the number of participants in the cohort [43]. Different neurotransmitter receptors and transporters from the same category were averaged to generate a corresponding neurotransmitter system map. The five neurotransmitter system maps were assigned to 246 regions, and the averaged value of all voxels within the region was defined as the region’s neurotransmitter value. After each neurotransmitter map z-normalized across 246 regions, we used a multivariate linear regression model to explore the contributions of neurotransmitter systems to the SA-related ANM in depression. The *relaimpo* (relative importance of regressor in linear models, version 2.2-5) package in R was used. Relative importance metrics can be used to address linear regression with multiple collinear regression [45]. The model was defined as follows:

$${Ov}=	 \, {\beta }_{0}+{\beta }_{1}\times {Dopamine}+{\beta }_{2}\times {GABA}+{\beta }_{3}\times {Glutamate}+{\beta }_{4}\\ 	 \times {Noradrenaline}+{\beta }_{5}\times {Serotonin}+\epsilon,$$

where *Ov* (overlap value) is the values of 246 ROIs from the SA-related ANM in depression. After determining the percentage of explained variance from these predictors, we assessed the relative contribution of each neurotransmitter system using bootstrapping. The multiple comparisons were corrected by FDR with *P* < 0.05.

### NeuroSynth meta-analysis

The SA-related ANM in depression was decoded by the NeuroSynth database (https://www.neurosynth.org) to identify the relevant cognitive functions [46, 47]. A public python code (https://github.com/NeuroanatomyAndConnectivity/gradient_analysis/blob/master/05_metaanalysis_neurosynth.ipynb) was used to perform this analysis [48]. Relevant cognitive functions were identified using FDR correction (*P* < 0.05).

## Results

### The SA-related ANM in depression

**Fig. 1:**
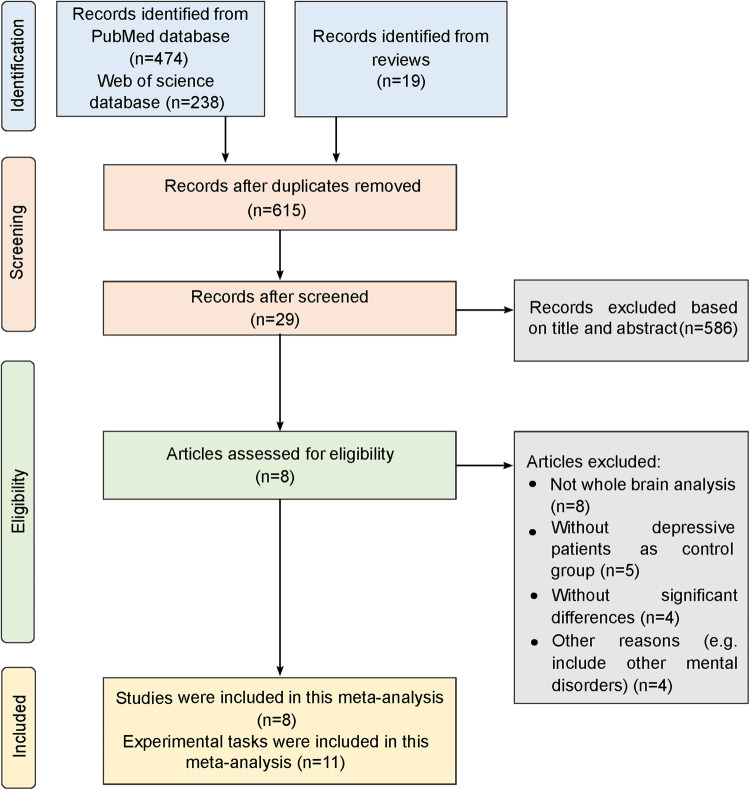
The PRISMA selection diagram of task-related neuroimaging studies in depression with SA.

**Fig. 2:**
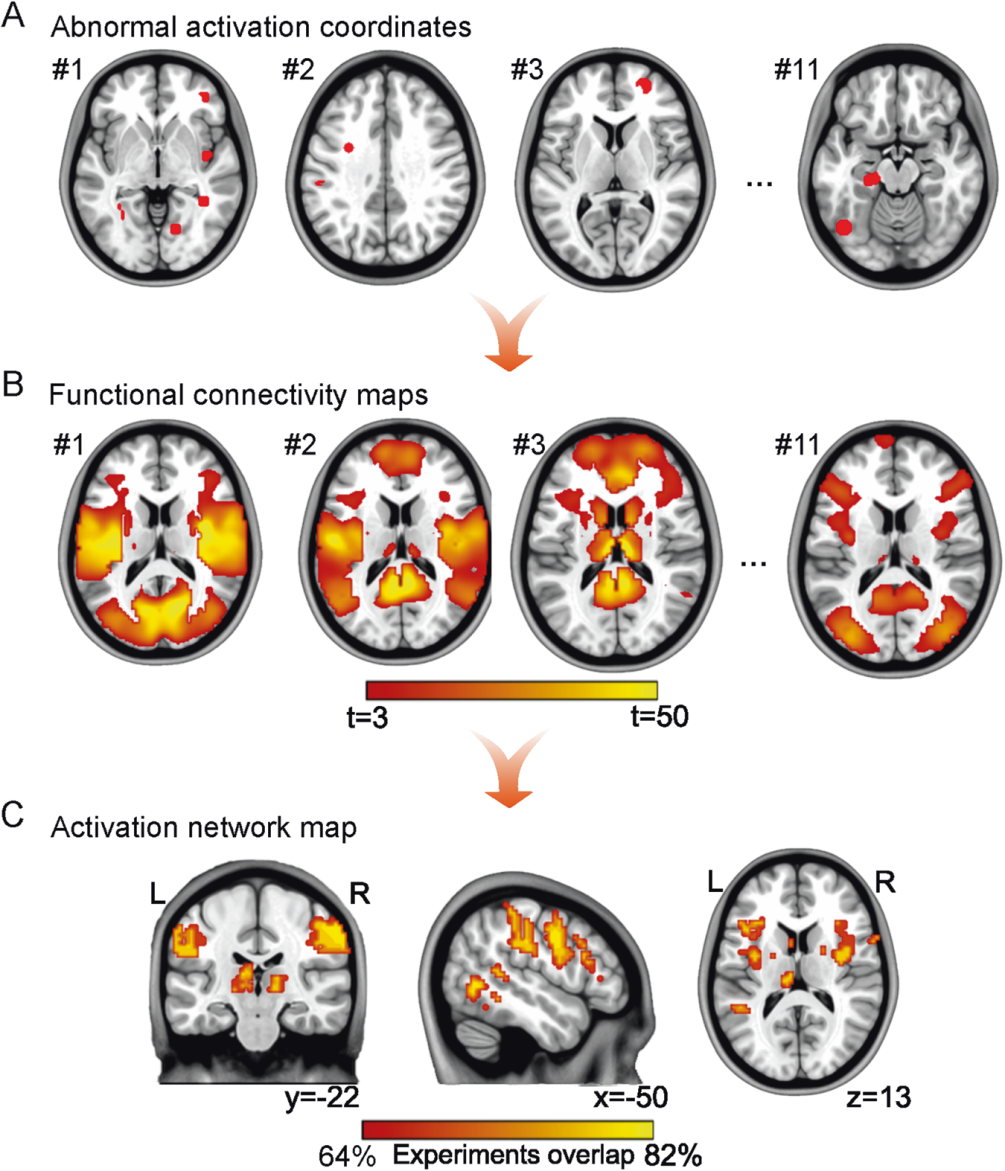
Network localization of heterogeneous activations in depression with SA. **A** Four-millimeter spheres centered on abnormal coordinates reported in each experimental task were created and combined to create a combined seed. **B** Brain regions are significantly connected to each combined seed across a normative connectome (*n* = 1000). All 1000 maps for each experimental task were compared against zero using a voxel-wise one-sample *t* test and with a threshold of *t* > 3. **C** Binarized (t = 3) t maps from all experimental tasks were overlapped to identify the regions connected to most of the combined seeds.

We identified eight studies with 11 experimental tasks that reported abnormal activations in depressive patients with SA (total *n* = 147) compared to depressive patients without SA (total *n* = 196) (Fig. 1; Table 1). Brain regions with abnormal activation from these studies were highly heterogeneous, but the majority (7/11, 64%) of combined seeds, generated by the abnormal activation coordinates (Supplementary Table 1) from the same experimental task, connected to the somato-cognitive action network (SCAN). The amygdala, left hippocampus, and right orbitofrontal and left temporal cortices were also included in this network map (Fig. 2). The identified SA-related ANM in depression was robust to parameter selection of threshold values in the *Overlapping section* (Dice coefficient = 0.62; Supplementary Fig. 1A) and seed sizes in the *RSFC analysis section* (6 mm: Dice coefficient = 0.42; 8 mm: Dice coefficient = 0.47; 10 mm: Dice coefficient = 0.49; Supplementary Fig. 1B). Using the leave-one-experiment-out approach, we verified that the identified SA-related ANM in depression is stable between all 11 experimental tasks and the maps with one experimental task removed, showing moderate to high similarity (Dice coefficient = 0.49–0.80; Supplementary Fig. 1C).

**Table 1:** Experimental tasks and clinical information of subjects in included studies.

First author (year)	Experimental task	Number of subjects			Mean age (years)			BDI			HDRS		
		MDD+SA N=147	MDD N=196	HC N=164	MDD+SA	MDD	HC	MDD+SA	MDD	HC	MDD+SA	MDD	HC
Jollant et al. (2008) [9]	Happy face; Angry face	13	14	16	40.3	43.9	32.4	7.2	8.1	2.6	2.4	2.9	1.1
Pan et al. (2011) [11]	Go/NoGo	15	15	14	16.2	15.9	15.2	15.5	4.4	2.1	N.A.	N.A.	N.A.
Pan et al. (2013a) [10]	Happy face; Angry face	14	15	15	16.2	15.9	15.3	15.1	4.4	1.9	N.A.	N.A.	N.A.
Pan et al. (2013b) [12]	Iowa Gambling Task (IGT)	15	14	13	16.2	15.8	15.2	15.5	4.6	2.2	N.A.	N.A.	N.A.
Vanyukov et al. (2016) [13]	Discounting task of value difference; Discounting task of tracking value of choices with longer versus shorter delays	13	13	22	70.4	73.4	72.1	N.A.	N.A.	N.A.	14.5	13.2	2.5
Olie et al. (2017) [14]	Cyberball Game	36	41	28	39.5	37.6	38.9	3.5	3	0	3.5	2	2
Ai et al. (2018) [8]	Faces task	18	54	26	37.7	37.6	39.0	N.A.	N.A.	N.A.	N.A.	N.A.	N.A.
Ji et al. (2021) [7]	Balloon Analog Risk Task (BART)	23	30	30	21.4	23.4	20.6	35.2	33.2	3.7	N.A.	N.A.	N.A.

*BDI* Beck Depression Inventory score, *HDRS* Hamilton Depression Rating Scale (17-item) score, *MDD+SA* major depressive disorder patients with suicide attempts, *MDD* major depressive disorder patients without suicidal attempts, *HC* healthy controls, *NA* not available.

### Specificity of the SA-related ANM in depression

**Fig. 3:**
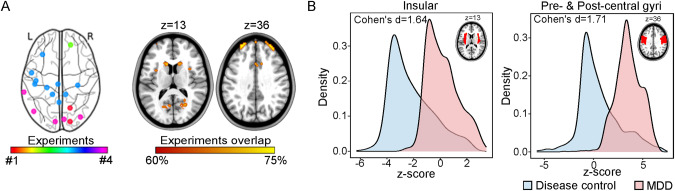
The specificity of SA-related ANM in depression. **A** Left: Combined abnormal activation coordinates across all experimental tasks of the disease control group. Coordinates from the same experimental task are shown with the same color. Right: SA-related ANM in the disease control group. Brain regions connected to over 60% of the combined seeds are shown in two specific brain slices. **B** Differences were calculated with Cohen’s d test in the priori insula and the post hoc precentral and postcentral gyri. At the upper right corner of the two density difference maps, the AAL3 mask of the corresponding brain regions is shown.

To determine the specificity of SA-related ANM in depression, we identified three studies with four experimental tasks in the disease control group (Supplementary Fig. 2 and Fig. 3; Supplementary Table 2), which reported the abnormal activation coordinates in BD and SCZ patients with SA (total *n* = 52) compared to patients without SA (total *n* = 81) (Fig. 3A; Supplementary Table 3). We found that the SA-related ANM in depression exhibited stronger connectivity in the prior-defined insula and post-hoc ROIs of precentral and postcentral gyri than the disease control group (Fig. 3B).

### Molecular mechanisms of the SA-related ANM in depression

**Fig. 4:**
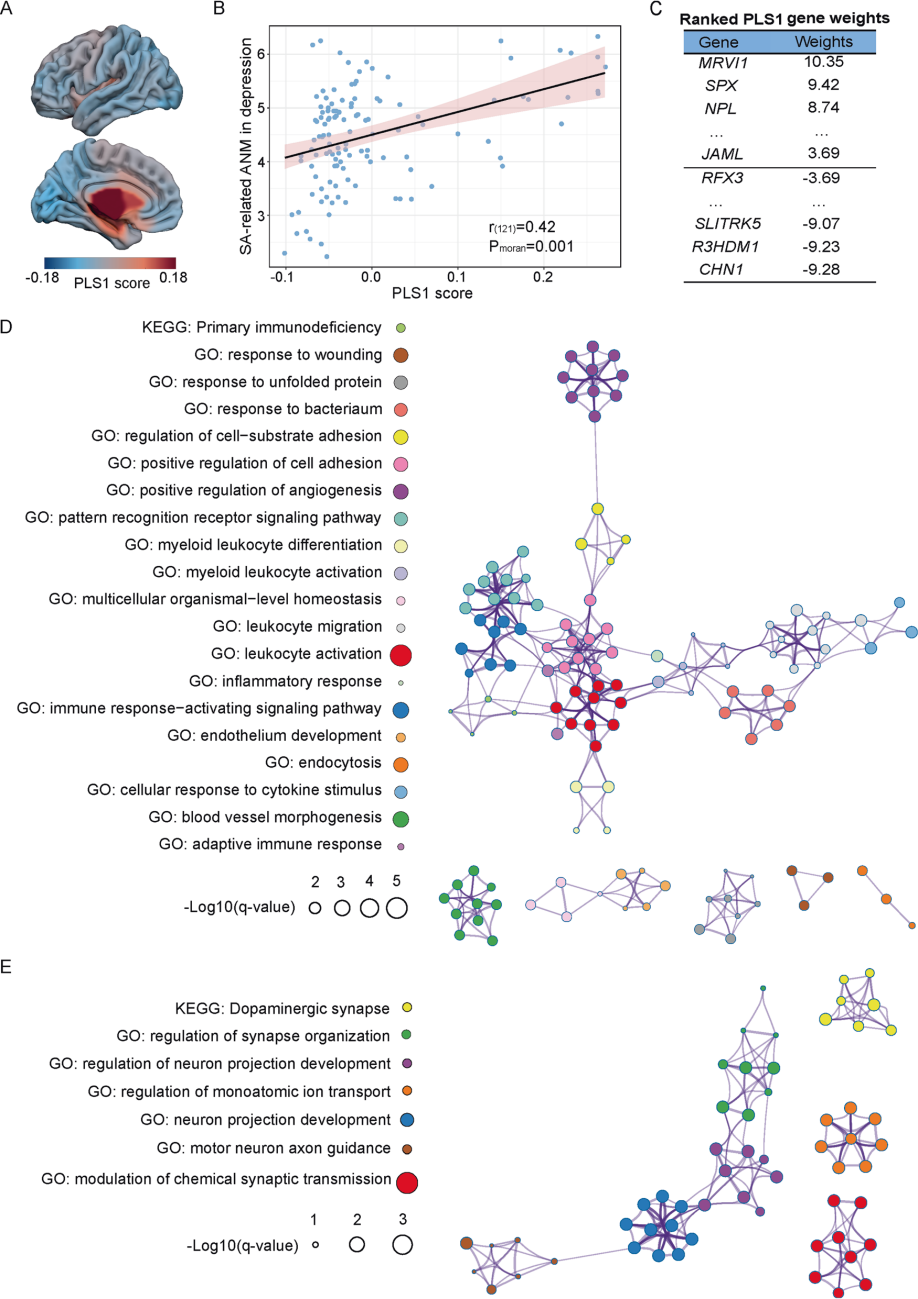
Gene PLS analysis and enrichment analyses. **A** Spatial map of PLS1 score in the left hemisphere. **B** The spatial correlation between the PLS1 score and the SA-related ANM in depression. **C** The ranked gene weights of PLS1 (*P*_FDR_ < 0.001). **D** Left: The significantly enriched biological processes (*P*_FDR_ < 0.05) of the PLS1+ gene set (q-value: the value from the Benjamini–Hochberg procedure for multiple comparison correction; color of the circle: different biological processes corresponding to the nodes of the enrichment network in the right). Right: Metascape enrichment network representing the similarities within each cluster and between clusters (circle nodes represent enriched biological processes; their size depends on the proportion of genes related to the enriched biological process among all input genes; nodes with the same color belong to the same cluster; nodes within the same cluster are typically similar to each other; biological processes with similarity >0.3 are connected through edges). **E** Left: The significantly enriched biological processes (*P*_FDR_ < 0.05) of the PLS1− gene set. Right: Metascape enrichment network representing the similarities within each cluster and between clusters.

We next performed transcriptomic analysis to determine which genes were associated with the SA-related ANM in depression. We found that the PLS1 explained 17.63% variance of the SA-related ANM in depression (Fig. 4A), which was significantly more than expected by chance (*P*_moran_ = 0.03). The spatial distribution of PLS1 was positively correlated with the SA-related ANM in depression (Fig. 4B; r_(121)_ = 0.42, *P*_moran_ = 0.001). This positive correlation means that genes positively (negatively) weighted on PLS1 are overexpressed in the brain regions with higher (lower) overlap [42, 49]. After correcting the normalized weights of PLS1, we found that 1458 positive weighted genes (PLS1+ gene set) and 1444 negative weighted genes (PLS1− gene set) (Fig. 4C; *P*_FDR_ < 0.001) remained. The PLS1+ gene set is mainly enriched in the inflammatory and immune biological processes, such as “leukocyte activation”, “immune response-activating signaling pathway”, and “primary immunodeficiency” (Fig. 4D). The PLS1− gene set is mainly enriched in the neuro projection- and synapse-related biological processes, such as “modulation of chemical synaptic transmission”, “regulation of synapse organization”, “motor neuron axon guidance”, and “dopaminergic synapse” (Fig. 4E).

**Fig. 5:**
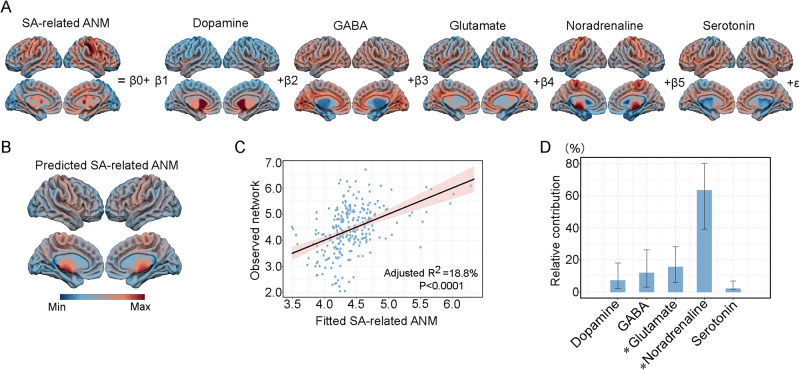
The relationships between different neurotransmitter systems and the SA-related ANM in depression. **A** A multiple linear regression model was used to determine the relationships between different neurotransmitter systems and the SA-related ANM in depression. **B** The predicted SA-related ANM in depression. **C** A scatterplot was generated to display the similarity between the fitted network and the observed SA-related ANM in depression. **D** The relative contributions of each neurotransmitter system were assessed using the *relaimpo* R package. Error bar, 95% bootstrap confidence intervals. An asterisk represents that *P* values survived after FDR correction with *P* < 0.05.

To further decode the SA-related ANM in depression by chemo-architectures, we investigated the relationship between neurotransmitter systems and the SA-related ANM in depression using a multiple linear regression model (Fig. 5A). The model explained 18% of the variance in the SA-related ANM in depression (F_(5240)_ = 11.5, *P* < 0.001, adjusted R^2^ = 0.18; Fig. 5B, C). The noradrenaline and GABA systems significantly contributed to the SA-related ANM in depression (noradrenaline weight = 0.33, *P*_FDR_ < 0.001; GABA weight = −0.24, *P*_FDR_ = 0.03). Notably, the noradrenaline system had the highest relative contribution (relative contribution = 63.5%; Fig. 5D).

### Cognitive functions related to SA in depression

**Fig. 6:**
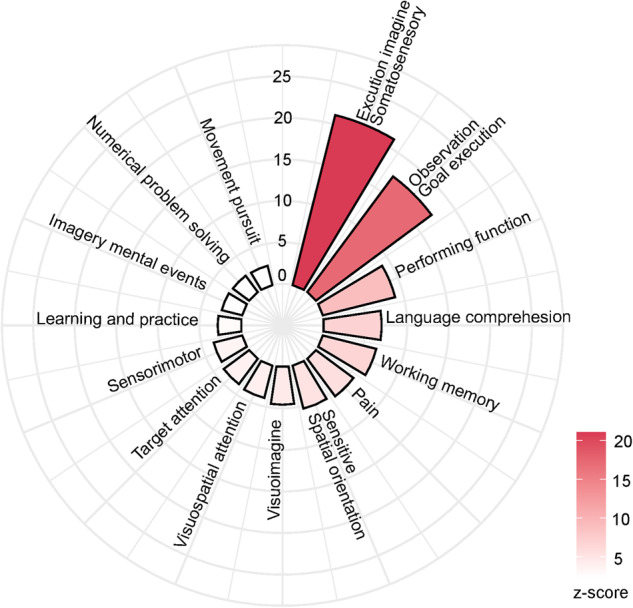
The NeuroSynth meta-analysis of the SA-related ANM in depression. The terms shown here are significantly associated with the SA-related ANM in depression.

We binarized the SA-related ANM in depression with a 60% threshold to generate a mask for NeuroSynth meta-analysis. We found fifteen significantly related cognitive functions (Fig. 6; *P*_FDR_ < 0.05). The three most relevant cognitive functions were “execution imagine and somatosensory” (z = 21.1), “observation and goal execution” (z = 17.3), and “performing function” (z = 8.9).

## Discussion

Using the recently proposed ANM technique, we found that heterogeneous neural activations were functionally connected to the SCAN in depression with SA. The identified SA-related ANM in depression was robust to the parameter perturbations and leave-one-experiment-out analyses, and was specific for depression. In the transcriptomic and chemo-architectures analyses, we revealed that genes significantly related to the SA-related ANM in depression were mainly enriched in inflammatory and immunity-related biological processes. The spatial distributions of noradrenaline and GABA systems recapitulated the robust SA-related ANM in depression. These findings provide new insight into unifying heterogeneous neuroimaging studies, further advancing our integrative understanding of psychopathology in depression with SA.

The heterogeneous brain regions with abnormal activation in depressive patients with SA across a series of experimental tasks were localized to a common brain network using the ANM approach. This finding might help us to explain the low reproducibility of functional neuroimaging studies. We found that the ANM result exhibited a larger experimental contribution (73%) than the ALE result (27%) to the consistent finding in the left insula (Supplementary Materials; Supplementary Fig. 4). This finding is consistent with numerous studies on network localization of neuropsychiatric symptoms [50], neurodegenerative diseases [28], and emotion processing [23]. The identified SA-related ANM shows robustness against parameter perturbations and specificity for other spectrum disorders (depression patients with SA vs. SCZ and BD patients with SA), suggesting that ANM may be one of the methods to resolve the low reproducibility across functional neuroimaging studies. Specifically, our findings show that regions located outside the abnormal activation loci, but functionally connected to these areas, can also be involved in the depressive process. This finding is consistent with a growing literature suggesting that symptoms localize to connected brain circuits, not to single brain abnormalities [19, 26, 51]. Furthermore, brain lesions caused depression and depression-relieving neuromodulation sites functionally link to a brain network that is comparable across 14 different datasets, suggesting therapeutic utility [52].

Heterogeneous activation findings in depressive patients with SA mainly converged on the SCAN which includes the motor area (M1), supplementary motor area (SMA), thalamus, posterior putamen, and postural cerebellum, and functionally connected to the dorsal anterior cingulate cortex (dACC), parietal cortex, and insula [53]. The SCAN plays an important role in the integration of mind and body, which has been argued to be crucial in psychology. The SMA, M1, insula, and parietal cortex in the SCAN are most often involved in response inhibition, which is the ability to suppress unneeded and unsafe actions or thoughts [54]. Abnormalities of these brain regions can lead to deficient inhibitory control (impulsivity), which may be an important factor in patients with SA [55]. Structural and functional abnormalities in the M1, insula, and parietal cortex were found in depressive patients with SA [10, 56, 57]. In addition, reminiscing on painful memories might harm response inhibition [58]. Overwhelming sad and painful memories can encourage rumination and mind-wandering to compete for limited cognitive resources [58], influencing the ability to stop or suppress suicidal behavior. Ji et al. found that individuals with depression were affected by a solid motivation to evade psychological pain [7]. This motivation can potentially result in suicide behavior by occupying brain resources, thereby disrupting the normal response inhibition function. In conclusion, dysfunction of the SCAN may play an important role in modulating emotion and cognition in depressive patients with SA.

The strongest peak of the SA-related ANM in depression was observed in the thalamus that integrates sensory input from the external environment [59]. The thalamus is crucial for processing information after stress exposure and may act as an interface for the stress response in the body and mind [60]. Suicidal people were shown to have an enlarged thalamus [61] and to suffer from stress dysregulation of the hypothalamus–pituitary–adrenal (HPA) axis [62]. In addition, the SA-related ANM in depression included the somatosensory cortex, amygdala, left hippocampus, and right orbitofrontal and left temporal cortices, which also have great significance in depression with SA. The amygdala, left hippocampus, and right orbitofrontal cortex are essential areas of the limbic system, which is generally known to be involved in emotion regulation and is implicated in depression and suicidal behavior [63, 64]. The somatosensory cortex is involved in body awareness and complex experience of pain, and it is less activated in response to pain stimuli [65]. Pain symptoms in patients with depression occupied a high rate (50%) based on a large-scale community study [66], which is significantly associated with lifetime suicide attempts [67]. Furthermore, a mega-analysis revealed that the organization of cortical networks involved in processing sensory information might be a more stable neuroimaging marker for depression than previously assumed alterations in higher-order neural networks like default mode and frontoparietal control networks [68].

Our transcriptional analysis revealed that genes associated with the SA-related ANM in depression were enriched in several biological processes, such as inflammatory and immune processes. A growing body of evidence indicates that inflammation plays an essential role in individuals with depression and SA [69]. The pro-inflammatory cytokines IL-1, IL-6, and TNF-α were found to be associated with behavioral changes in depressive patients [70]. Compared to the healthy control group, SA individuals displayed significantly different leukocyte levels, which are part of our immune system [71]. Our neurotransmitter system analysis revealed that the GABA and noradrenaline neurotransmitter systems were significantly correlated with the SA-related ANM in depression. GABA serves as the major inhibitory neurotransmitter in the brain, which improves mood, increases relaxation, and alleviates pain. Reduced GABA levels have been observed in postmortem tissues of depressive patients with SA [72]. Noradrenaline is associated with executive functioning and governs cognition, motivation, and intelligence. These three factors are crucial to social relationships, and their dysfunction affects the quality of life of depressive patients [73]. Noradrenaline may underly the pathophysiology of depression, and may be a biomarker for the treatment of depressive patients [74]. Furthermore, the relationships between GABA and noradrenaline neurotransmitters with SA-related ANM in depression were opposite, which may be related to the distinct roles in HPA axis activation. The GABA and noradrenaline can separately inhibit and excite the hypothalamic activation of the HPA axis [62]. The HPA axis is abnormally hyperactivated in depressive patients with SA [62]. These transcriptomic and chemo-architecture analyses possibly demonstrated the molecular mechanism of the SA-related ANM in depression. In addition to micro- and meso-associations with SA-related ANM in depression, we also found behavioral relevance of SA-related ANM, such as the execution function, memory, and attention. These cognitive functions were found to be impaired in depressive patients [75, 76]. In particular, dysfunction of execution [77] and attention [78] was found to be more severe in depression patients with SA.

Several limitations need to be considered in our study. First, given that we used brain activation abnormalities as inputs rather than lesions, our results provide only correlational but not causational information [79]. The causation can be tested with prospective studies and brain stimulation studies [80]. Second, because of the limited brain activation investigation in depressive patients with SA, the number of studies included is relatively low, which may cause negative results. In addition, this study included various cognitive and emotional experimental tasks, which may increase the heterogeneity and impact the reproducibility and generalizability of the findings. However, we conducted the leave-one-experiment-out analysis to validate the stability of SA-related ANM in depression. Similar issues existed in the disease control group (i.e., schizophrenia and bipolar disorders). Thus, the specificity of SA-related ANM in depression should be described cautiously. Future studies can enroll more participants in the specific task to investigate the SA-related ANM in depression [23]. Finally, we explored the relationships between the SA-related ANM in depression and gene expression levels and neurotransmitter distributions of healthy controls. Future studies should collect the patients’ gene expression and neurotransmitter data in the brain, which can reveal the correlated directionality between micro- and meso-scale distributions and the SA-related ANM in depression.

In summary, we integrated the heterogeneous abnormal neural activations into the SCAN in depressive patients with SA using the ANM framework. The findings support the notion that body–mind integration is crucial to depressive patients with SA. The robust SA-related ANM can be decoded by transcriptomic and molecular profiles, possibly suggesting several important multiscale interactions in depression patients with SA.

## Supplementary information

Supplemental information

## Data Availability

Human gene expression data that support the findings of this study are available in the Allen Brain Atlas (https://human.brain-map.org/static/download). A publicly available resting-state connectome database was obtained from Brain Genomics Superstruct Project (https://dataverse.harvard.edu/dataverse/GSP). Neurotransmitter receptors and transporters were obtained from https://github.com/netneurolab/hansen_receptors/tree/main/data/PET_nifti_images.

## Code availability

Transcriptomic data was preprocessed by abagen toolbox (https://github.com/rmarkello/abagen). Human gene The gene enrichment analysis was conducted by Metascape (https://metascape.org/). Multiple linear-regression model was performed using the *relaimpo* in R package (http://prof.beuth-hochschule.de/groemping/software/relaimpo/).
