# Supplementary material for: Divergent suicidal symptomatic activations converge on somato-cognitive action network in depression
Source: Mol Psychiatry. 2024 Feb 14;29(7):1980–9. doi: 10.1038/s41380-024-02450-7 (PMC11408245; doi:10.1038/s41380-024-02450-7)
Supplement: Supplementary file 1 — Supplemental information [file 41380_2024_2450_MOESM1_ESM.docx]

**Divergent Suicidal Symptomatic Activations Converge on Somato-cognitive Action Network in Depression**

**Jiao Li^1,2,3🖂^ PhD, Dajing Wang^1,2,3^ MD, Jie Xia^1,2^ PhD, Chao Zhang^1,2^ MD, Yao Meng^1,2^ PhD, Shuo Xu^1,2^ MD, Huafu Chen^1,2🖂^ PhD, Wei Liao^1,2🖂^ PhD,**

^1^ The Clinical Hospital of Chengdu Brain Science Institute, School of Life Science and Technology, University of Electronic Science and Technology of China, Chengdu 611731, P.R. China.

^2^ MOE Key Lab for Neuroinformation, High-Field Magnetic Resonance Brain Imaging Key Laboratory of Sichuan Province, University of Electronic Science and Technology of China, Chengdu 611731, P.R. China.

^3^ These authors contributed equally: Jiao Li, Dajing Wang.

**^🖂^** Corresponding author:

Jiao Li (jiaoli@uestc.edu.cn), Huafu Chen (chenhf@uestc.edu.cn), Wei Liao (weiliao.wl@gmail.com). The Clinical Hospital of Chengdu Brain Science Institute, MOE Key Laboratory for Neuroinformation, University of Electronic Science and Technology of China, Chengdu 611731, P.R. China. Fax: +86-28-61831273. Tel: +86-28-61831273.

# Supplementary Methods

**Robustness analysis**

Additional analyses were performed to investigate the robustness of the identified SA-related ANM in depression:

1) In the *RSFC analysis section*, spheres with a 6-mm, 8-mm, and 10-mm radius centred at reported coordinates were additionally generated to investigate the influence of radius parameters on SA-related ANM in depression. Dice coefficients were computed between the main result and the three validation maps.

2) In the *overlapping section*, the statistical threshold was also set at t > 5 (corresponding to voxel-wise FDR correction P < 10^−5^) to test whether threshold selection would influence the SA-related ANM in depression.

3) Leave-one-experiment-out analysis was performed to explore the influence of perturbation of experimental task selection. Dice coefficients were measured between SA-related ANM maps with all experimental tasks and the maps with one experimental task removed.

**Activation likelihood estimation(ALE) analysis**

We utilized the GingerALE3.0.2 (http://www.brainmap.org/ale/) to perform an activation likelihood estimation(ALE) meta-analysis on abnormal activations in depressive patients with SA. The fundamental principles of the ALE algorithm have been described in previous studies^1, 2^. First, we created a 3D Gaussian kernel of each individual coordinate by a full-width at half-maximum value according to the sample size from each experimental task. Second, we calculated the modeled activation map for each experimental task, and then obtained the ALE map on a voxel-by-voxel level by combining these individual modeled activation maps. Finally, to test whether the ALE map could be effected by random coordinate distributions, we compared the ALE map to a null distribution that was generated by a permutation test (1,000 permutations). A non-parametric *P* value map was generated. For this result, a cluster-forming threshold was set at P<0.01 and a cluster-level Family-Wise Error (FWE) correction threshold at P<0.05.

#
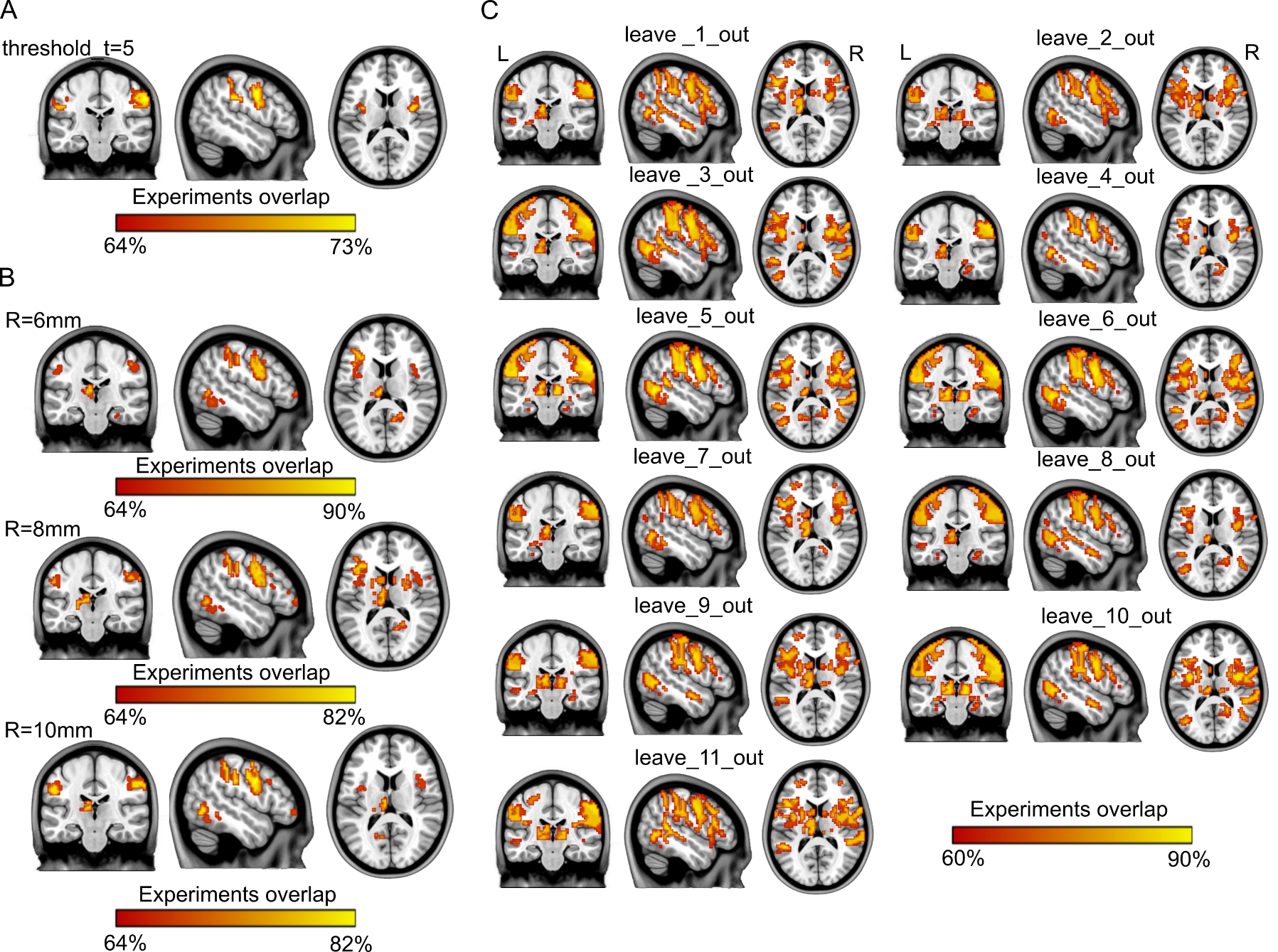
Supplementary Results

**Supplementary Figure 1. Robustness of SA-related ANM in depression.** (A) The network was identified with the experiment-level t-map thresholded at t>5. (B) Networks were identified with the seed sphere at 6-mm, 8-mm, and 10-mm. (C) Networks were identified using the leave-one-experiment-out test.


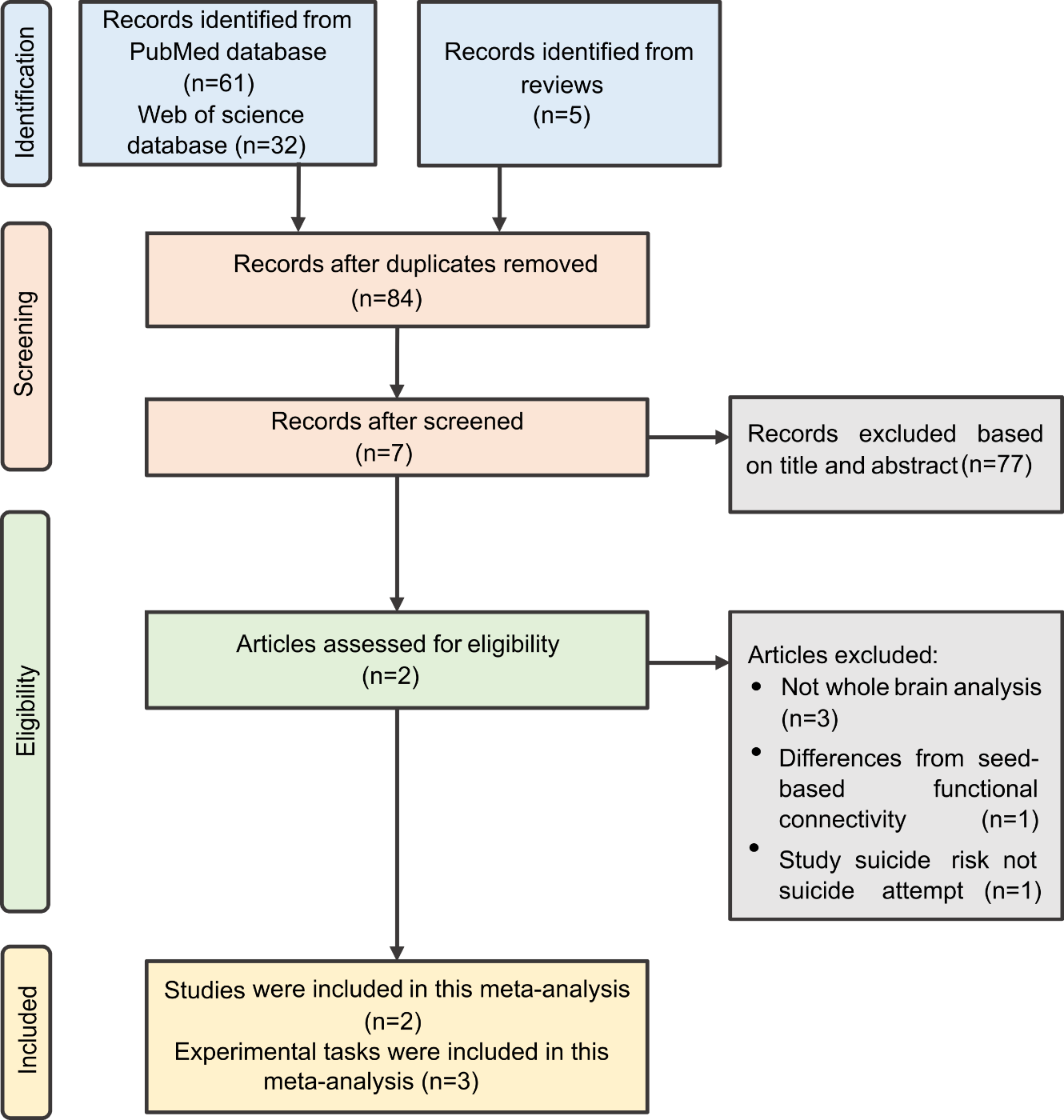


**Supplementary Figure 2**. The PRISMA selection diagram of task-related neuroimaging studies in BD with SA.


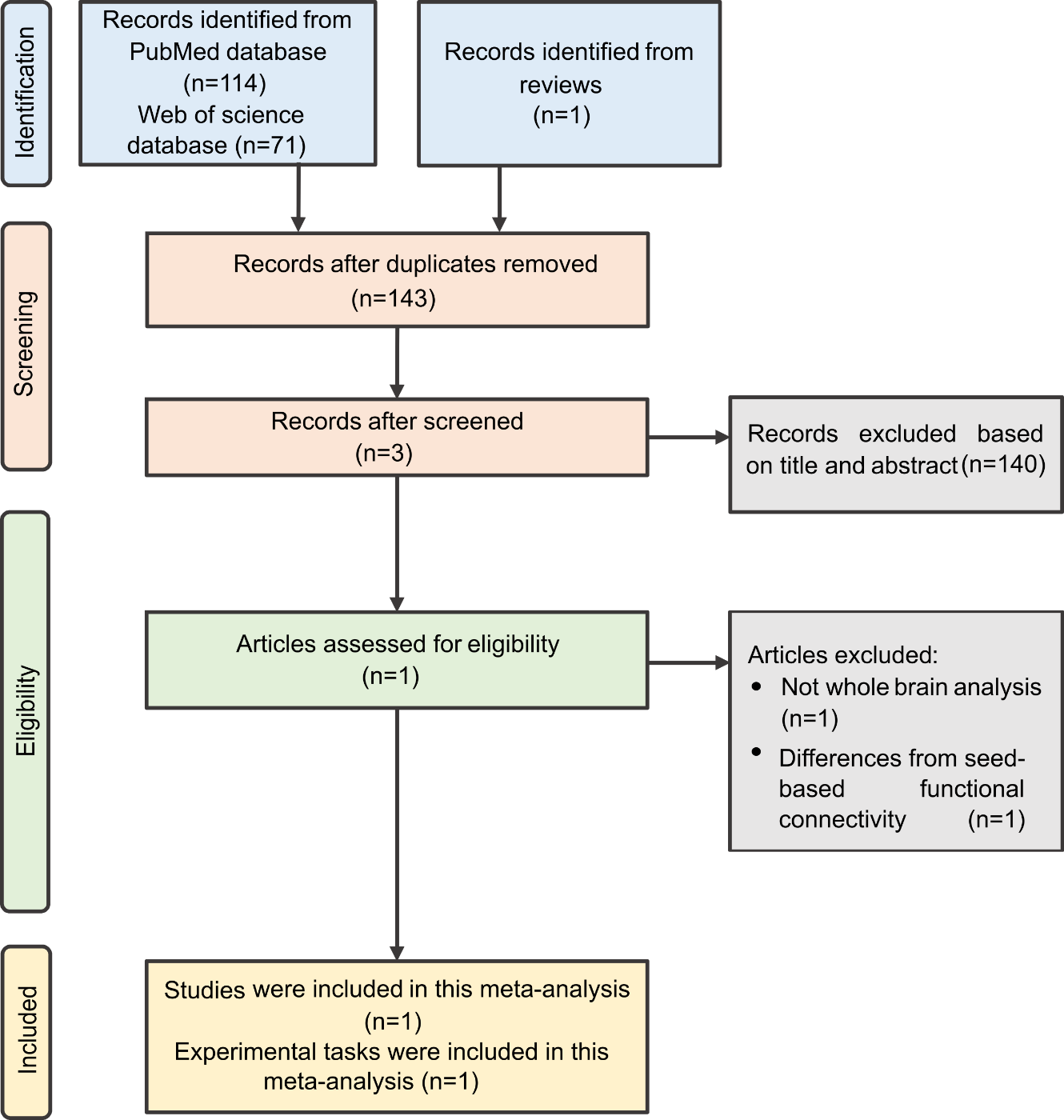


**Supplementary Figure 3**. The PRISMA selection diagram of task-related neuroimaging studies in SCZ with SA.


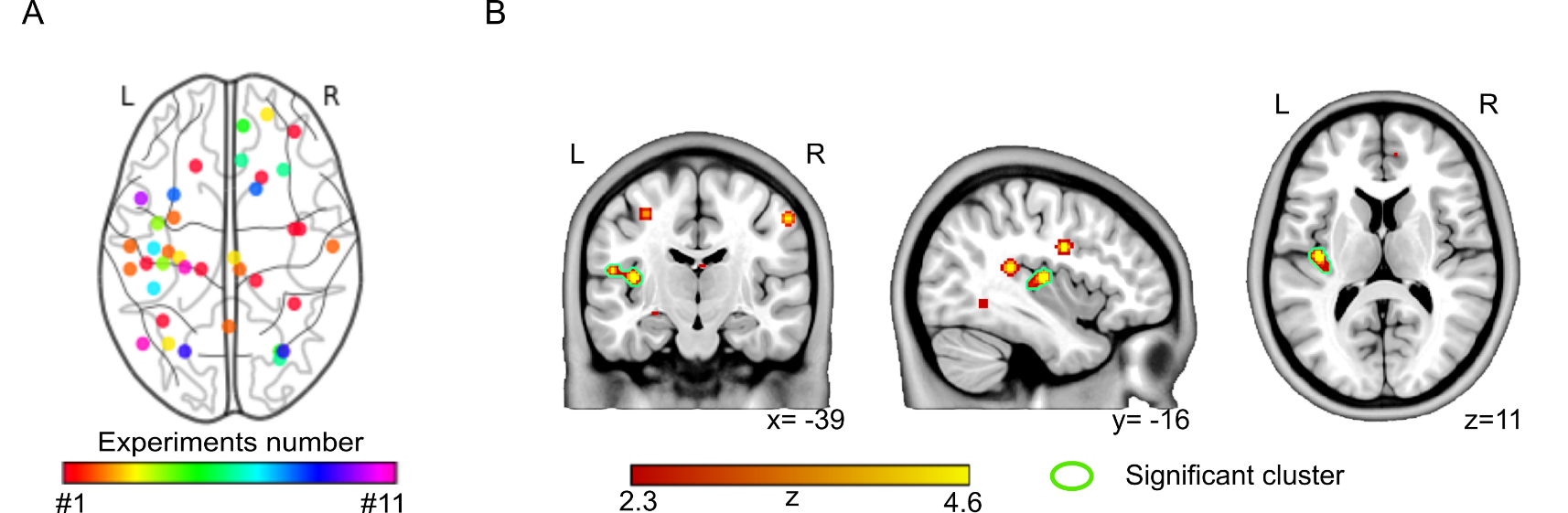


**Supplementary Figure 4. The ALE map of depression with SA.** (A) The combined abnormal activation locations of all coordinates across all experimental tasks of depression with SA, coordinates with the same experimental task were shown with the same color. (B) The regions with a z-score over 2.3 were shown on the brain template, and the cluster within the green circle was significant (cluster-FWE correction P<0.05).

The ALE result has three contributors as follows:

Contributors to cluster #1

1 foci from Pan et al., 2013: happy face

1 foci from Ji et al., 2021: dynamic decision making balloon analogue risk task

1 foci from Olie et al., 2017: Cyberball game

**Supplementary Table 1**. Coordinates and corresponding brain regions from studies in depression with SA.

| **Article** | **Experimental task** | **Coordinates** | **Brain region** |
| --- | --- | --- | --- |
| Jollant et al. (2008)^3^  Talairach coordinates | Angry face | (x=29, y=19, z=-24) | Lateral orbitofrontal cortex |
|  |  | (x=29, y=-74, z=-18) | Cerebellum_R |
|  |  | (x=7, y=33, z=53) | Superior frontal gyrus_R |
|  | Happy face | (x=29, y= -70, z=-18) | Cerebellum_R |
|  |  | (x=7, y=44, z=4) | Anterior cingulate gyrus_R |
| Pan et al. (2011)^4^  MNI coordinates | Go/No_Go | (x=15, y=15, z=42) | Anterior cingulate gyrus_R |
|  |  | (x=-30, y=12, z=18) | Insula_L |
| Pan et al. (2013a)^5^  MNI coordinates | Angry face | (x=18, y=21, z=42) | Anterior cingulate gyrus_R |
|  |  | (x=36, y=45, z=-9) | Ventromedial prefrontal cortex_R |
|  |  | (x=15, y=-33, z=54) | Primary sensory cortex_R |
|  |  | (x=-15, y=-27, z=63) | Primary sensory cortex_L |
|  |  | (x=-18, y=27, z=27) | Dorsolateral prefrontal cortex_L |
|  |  | (x=-45, y=-24, z=39) | Superior parietal gyrus_L |
|  |  | (x=39, y=-6, z=-9) | Middle temporal gyrus_R |
|  |  | (x=36, y=-45, z=-9) | Fusiform gyrus_R |
|  |  | (x=12, y=-69, z=3) | Occipital cortex_R |
|  |  | (x=-36, y=-54, z=-6) | Fusiform gyrus_L |
|  |  | (x=36, y=-6, z=18) | Insula_R |
|  | Happy face | (x=0, y=-57, z=36) | Posterior cingulate gyrus_L |
|  |  | (x=-54, y=-15, z=15) | Superior temporal gyrus_L |
|  |  | (x=-54, y=-27, z=36) | Inferior parietal gyrus_L |
|  |  | (x=6, y=-27, z=21) | Thalamus_R |
|  |  | (x=-33, y=-18, z=51) | Primary sensory cortex_L |
|  |  | (x=-30, y=0, z=36) | Supplementary motor area_L |
|  |  | (x=57, y=-15, z=48) | Superior parietal gyrus_R |
| Pan et al. (2013b)^6^  MNI coordinates | Iowa Gambling Task (IGT) | (x=-33, y=-66, z=30) | Middle temporal gyrus_L |
|  |  | (x=-27, y=-21, z=-12) | Hippocampus_L |
|  |  | (x=21, y=54, z=3) | Medial frontal gyrus_R |
|  |  | (x=3, y=-21, z=18) | Thalamus_R |
| Vanyukov et al. (2016)^7^  MNI coordinates | Discounting task of value difference | (x=-48, y=10, z=35) | Dorsolateral prefrontal cortex_L |
|  | Discounting task of tracking value choices with longer vs shorter delay | (x=-24, y=-26, z=-16) | Parahippocampal gyrus_L |
|  |  | (x=-47, y=-66, z=-13) | Middle occipital gyrus_L |
| Olié et al. (2017)^8^  Talairach coordinates | Cyberball game | (x=-39, y=-36, z=18) | Supramarginal gyrus_L |
|  |  | (x=-39, y=-16, z=13) | Posterior insula |
| Ai et al. (2018)^9^  MNI coordinates | Faces task | (x=30, y=-70, z=-8) | Fusiform gyrus extending to lingual gyrus_R |
|  |  | (x=-24, y=-70, z=-11) | Fusiform gyrus extending to lingual gyrus_L |
| Ji et al. (2021)^10^  MNI coordinates | Balloon Analogue Risk Task (BART) | (x=-36, y=-24, z=6) | Insular cortex_L |
|  |  | (x=-39, y=-3, z=30) | Dorsolateral frontal cortex_L |

MNI, Montreal Neurological Institute.

**Supplementary Table 2**. Enrolled experimental tasks and clinical information of BD and SCZ patients with SA.

| **First author**  **（year）** | **Experimental task** | **Subject number** | | | **Mean age** | | | **Scale** | | | | |
| --- | --- | --- | --- | --- | --- | --- | --- | --- | --- | --- | --- | --- |
|  |  | Patients with SA  N=52 | Patients without SA  N=81 | HC  N=43 | Patients with SA | Patients without SA | HC | Patients with SA | Patients without SA | HC | Patients with SA | Patients without SA |
| **BD** | | | | | | | | | | | | |
|  | |  | | |  | | | MADRS | | |  | |
| Shaffer et al.  (2022)^11^ | Flashing checkerboard task | 19 | 20 |  | 46.8 | 31.0 |  | 1.37 | 0.35 |  |  |  |
| **SCZ** | | | | | | | | | | | | |
|  |  |  | | |  | | | BIS total | | | PANSS | |
| Potvin et al.  (2018)^12^ | Balloon Analogue Risk Task of Inflation event;  Balloon Analogue risk task of success event | 13 | 19 | 21 | 39.1 | 32.1 | 32.1 | 67.0 | 63.7 | 52.9 | Positive 17.0  Negative 16.2  General 37.1 | Positive 16.1  Negative 17.5  General 35.7 |
|  |  |  |  |  |  |  |  | MACVI | | | PANSS | |
| Athanassiou et al. (2021)^13^ | Emotional processing task (angry faces) | 20 | 42 | 22 | 18-60 | 18-60 | 18-60 | Violence 1.8  Victimization 0.5 | Violence 1.8  Victimization 0.6 |  | Positive 18.3  Negative16.8  Disorganized 12.3  Affect 7.4  Hostility 8.8 | Positive 15.5  Negative16.7  Disorganized 11.0  Affect 6.2  Hostility 6.7 |

MADRS, Montgomery- Asberg Depression Rating Scale; BIS, Barratt Impulsiveness Scale; PANSS, Positive and Negative Syndrome Scale; MACVI, The MacArthur Community Violence Instrument.

**Supplementary Table 3**. Coordinates and corresponding brain regions from studies in BD and SCZ with SA

| **Article** | **Experimental task** | **Coordinates** | **Brain region** |
| --- | --- | --- | --- |
| **BD** | | | |
| Shaffer et al. (2022)^11^  MNI coordinates | Flashing checkerboard task | (x=-9, y=-88, z=34) | Medial & Lateral Occipital Cortex |
|  |  | (x=-48, y=-70, z=-26) | Cerebellar Lobule V, VI_L |
|  |  | (x=7, y=-84, z=37) | Superior Occipital Cortex_R |
|  |  | (x=37, y=-80, z=-27) | Cerebellar Lobule VI_R |
|  |  | (x=-66, y=-36, z=14) | Superior Temporal Gyrus_L |
| **SCZ** | | | |
| Potvin et al. (2018)^12^  Talairach coordinates | Balloon Analogue Risk Task of Inflation event | (x=18, y=-64, z=-20) | Cerebellar declive_R |
|  |  | (x=18, y=-85, z=1) | Lingual gyrus_R |
|  | Balloon Analogue Risk Task of success event | (x=18, y=44, z=7) | Medial frontal gyrus/anterior cingulate gyrus_R |
| Athanassiou et al. (2021)^13^  MNI coordinates | Faces task: angry faces vs. control | (x=-4, y=-22, z=40) | Median cingulate gyrus_L |
|  |  | (x=-32, y=30, z=46) | Middle frontal gyrus_L |
|  |  | (x=-2, y=-54, z=54) | Precuneus_L |
|  |  | (x=-14, y=-40, z=6) | Hippocampus_L |
|  |  | (x=32, y=-24, z=70) | Primary motor cortex_R |
|  |  | (x=-44, y=-16, z=46) | Primary motor cortex_L |
|  |  | (x=10, y=-40, z=-18) | Cerebellum |
|  |  | (x=-52, y=-2, z=26) | Precentral area_L |
|  |  | (x=-46, y=-22, z=14) | Rolandic operculum_L |
|  |  | (x=-24, y=-28, z=60) | Primary motor cortex_L |

# References

1. Eickhoff SB, Bzdok D, Laird AR, Kurth F, Fox PT. Activation likelihood estimation meta-analysis revisited. *Neuroimage* 2012; **59**(3)**:** 2349-2361.

2. Eickhoff SB, Laird AR, Grefkes C, Wang LE, Zilles K, Fox PT. Coordinate-Based Activation Likelihood Estimation Meta-Analysis of Neuroimaging Data: A Random-Effects Approach Based on Empirical Estimates of Spatial Uncertainty. *Hum Brain Mapp* 2009; **30**(9)**:** 2907-2926.

3. Jollant F, Lawrence NS, Giampietro V, Brammer MJ, Fullana MA, Drapier D *et al.* Orbitofrontal cortex response to angry faces in men with histories of suicide attempts. *Am J Psychiat* 2008; **165**(6)**:** 740-748.

4. Pan LA, Batezati-Alves SC, Almeida JRC, Segreti A, Akkal D, Hassel S *et al.* Dissociable Patterns of Neural Activity During Response Inhibition in Depressed Adolescents With and Without Suicidal Behavior. *J Am Acad Child Psy* 2011; **50**(6)**:** 602-611.

5. Pan LA, Hassel S, Segreti AM, Nau SA, Brent DA, Phillips ML. Differential patterns of activity and functional connectivity in emotion processing neural circuitry to angry and happy faces in adolescents with and without suicide attempt. *Psychol Med* 2013a; **43**(10)**:** 2129-2142.

6. Pan L, Segreti A, Almeida J, Jollant F, Lawrence N, Brent D *et al.* Preserved hippocampal function during learning in the context of risk in adolescent suicide attempt. *Psychiat Res-Neuroim* 2013b; **211**(2)**:** 112-118.

7. Vanyukov PM, Szanto K, Hallquist MN, Siegle GJ, Reynolds CF, Forman SD *et al.* Paralimbic and lateral prefrontal encoding of reward value during intertemporal choice in attempted suicide. *Psychol Med* 2016; **46**(2)**:** 381-391.

8. Olié E, Jollant F, Deverdun J, de Champfleur NM, Cyprien F, Le Bars E *et al.* The experience of social exclusion in women with a history of suicidal acts: a neuroimaging study. *Sci Rep* 2017; **7**(1)**:** 89.

9. Ai H, van Tol MJ, Marsman JBC, Veltman DJ, Ruhe HG, van der Wee NJA *et al.* Differential relations of suicidality in depression to brain activation during emotional and executive processing. *J Psychiatr Res* 2018; **105:** 78-85.

10. Ji X, Zhao J, Li H, Pizzagalli DA, Law S, Lin P *et al.* From motivation, decision-making to action: An fMRI study on suicidal behavior in patients with major depressive disorder. *J Psychiatr Res* 2021; **139:** 14-24.

11. Shaffer JJ, Jr., Willour V, Fiedorowicz JG, Christensen GE, Long JD, Johnson CP *et al.* Distinct patterns of altered quantitative T1rho and functional BOLD response associated with history of suicide attempts in bipolar disorder. *Brain Imaging Behav* 2022; **16**(2)**:** 820-833.

12. Potvin S, Tikasz A, Richard-Devantoy S, Lungu O, Dumais A. History of Suicide Attempt Is Associated with Reduced Medial Prefrontal Cortex Activity during Emotional Decision-Making among Men with Schizophrenia: An Exploratory fMRI Study. *Schizophr Res Treatment* 2018; **2018:** 9898654.

13. Athanassiou M, Dumais A, Iammatteo V, De Benedictis L, Dubreucq JL, Potvin S. The processing of angry faces in schizophrenia patients with a history of suicide: An fMRI study examining brain activity and connectivity. *Prog Neuropsychopharmacol Biol Psychiatry* 2021; **107:** 110253.
